# Biological Diversity Associated with Pesticides Residues in Certain Egyptian Watercourses

**DOI:** 10.1007/s00244-025-01129-6

**Published:** 2025-05-09

**Authors:** Asmaa Abdel-Motleb, Rania M. Abd El-Hamid, Sara S. M. Sayed

**Affiliations:** 1 Environmental Research Department, Theodor Bilharz Research Institute, Giza, Egypt; 2 Central Agricultural Pesticides Labratory, Agricultural Research Centre, Giza, Egypt

## Abstract

The aquatic environment has been contaminated by pyrethroids and triazole pesticide applications, which pose serious health risks to the aquatic ecosystem and human beings. Therefore, the current study aims to evaluate water quality parameters, fungal diversity, and distribution of snails and aquatic plants of certain Egyptian water courses contaminated with pyrethroids and triazole pesticides. Seasonal samples were taken throughout 2021 from different water courses at Giza Governorate and Tanta (Gharbeya Governorate). Qualitative and quantitative surveys showed significant differences in water physical parameters between the two investigated governorates. Deltamethrin, permethrin, Es-fenvalerate, and lambada-cyhalothrin showed the highest pyrethroids concentrations, while tebuconazole, tetraconazole, and difenoconazole were the highest triazole concentrations. Fungal diversity displayed 21 molecularly identified fungal species related to four fungal genera: *Aspergillus, Fusarium*, *Penicillium,* and *Trichoderma*. *Penicillium* sp. and *Aspergillus niger* were the most frequent species. Snail diversity recorded 10 and 9 species in Giza and Tanta, respectively. *Physa acuta* was the most abundant snail. Ten species of aquatic plants were observed in Giza, while six species were observed in Tanta. Specifically, *Eichhornia crassipes* and *Lemna gibba* were the dominant species in the two governorates, with the relative abundance (39 and 22%) in Giza and (27 and 23%) in Tanta, respectively. Water quality parameters and seasonal variations could control fungal diversity, snails, and aquatic plant distribution. Different relations between pesticides and biological communities may reflect the ability/inability of certain snails and fungi species to commensalism with pesticide concentrations. Continuous pesticide monitoring is essential for life below water and aligns with SDG14.

**Supplementary Information:**

The online version contains supplementary material available at 10.1007/s00244-025-01129-6.

## Introduction

The prime objective of most countries to increase the crop yield to meet the needs of the growing human population requires the widespread applications of pesticides, which protect the crop from pests and help to improve the quality and quantity of crops (Yadav et al. 2020). However, pesticide use harms the environment and affects non-targeted organisms and targeted pests, raising concerns that have persisted for decades (Ebrahim et al. 2020). Approximately, 1 to 2.5 billion tons of pesticides is used in farmland and towns every year around the world, which has become a significant source of chemical pollutants in the environment (Shefali et al. 2021). Pyrethroids and triazoles are widely used insecticides and fungicides in many countries (Singh et al. 2018).

Pyrethroid pesticides are a group of synthetic organic insecticides similar to the natural pesticide pyrethrum, which is produced by *Chrysanthemum* flowers. They have been used to control pests in agriculture, greenhouses, and veterinary facilities since the 1980s due to their high efficiency and low toxicity to vertebrates compared to the organophosphate and carbamate groups of pesticides (Yoo et al. 2016). Triazoles are aromatic heterocyclic organo nitrogen compounds with a vast application spectrum, varying from materials (polymers), agricultural fungicides, pharmaceuticals, photoactive chemicals, and dyes (Agalave et al. 2011). Although pyrethroids and triazoles permit the development of crop production by controlling diseases and pests, they may also have adverse effects through their long-term toxicity and persistence, making them eco-disrupting chemicals (Pennington et al. 2019), and lead to the loss of the floral and faunal diversity (Misaki et al. 2019). Pesticides can contaminate the aquatic environment through several routes, such as runoff, spray drift, and leaching of pesticides and pose serious health risks to the ecosystem. Also, they could contaminate the soil, groundwater, surface water, air, edible plants, and animals and affect human health (Aktar et al. 2009; Cui et al. 2021), resulting in directly affecting all levels of biological organization, including primary producers, microorganisms, invertebrates, and fish. So, many groups of pyrethroids and triazoles have been listed as chemical agents with high toxicological risk: carcinogenic, mutagenic, and teratogenic (Cavalier et al. 2023; Swathy et al. 2024). Continuous pesticide monitoring is essential for life below water and aligns with the Sustainable Development Goals (SDG14), which highlight water quality, biodiversity, and the health of aquatic organisms. The QuEChERS (Quick, Easy, Cheap, Effective, Rugged, and Safe) extraction method has gained popularity in multi-residue pesticide analysis, offering advantages over traditional methods (Abdel Ghani and Hanafi 2016).

Aquatic snails play a significant role as indicator organisms for biological monitoring and risk assessment strategies (Borcherding and Volpers 1994). Also, snail communities are influenced by the prevailing physicochemical parameters, which determine their abundance, occurrence, and seasonal variations (Pemola et al. (2015). These parameters include dissolved oxygen, pH, water temperature, physical nature of the substratum, depth, current velocity, and nutritive content of the water body (Rai and Jauhari 2016). So, it is important to investigate the environmental parameters that control snail distribution in the freshwater ecosystem. Also, macrophytes are used to assess the ecological status of watercourses (Tarkowska-Kukuryk and Grzywna 2022). Besides, they play a crucial role in the trophic structure of aquatic ecosystems, interacting with higher trophic levels by providing food and refuge for the macroinvertebrates and fish (Elosegi et al. 2018). In addition, macrophytes affect water quality through their involvement in nutrient cycling and sediment re-suspension (Kleeberg et al. 2010). In the aquatic environment, microbes behave as growers, users, and heterotrophic organisms and they are involved in nutrient cycling that maintains the ecosystem balance (Schoffelen et al. 2019; Zhang et al. 2021). For example, fungi act as decay agents, playing significant roles in bioremediation, nutrient cycling, and ecosystem functioning (Ortiz-Vera et al. 2018). Chemical pollutants such as insecticides, fungicides, and herbicides are destructive for freshwater fungi, macroinvertebrates, and aquatic plants, which are considered vital biomarkers of contamination and aquatic environmental health (Kusi et al. 2020; Wang et al. 2023). Few studies have quantitatively assessed the effect of freshwater pollutants on fungal diversity and community, although the fungal communities are known to respond to pollution (Zhou et al. 2020). Therefore, the current study of freshwater canals in Egypt provides data on monitoring the pyrethroid and triazole pesticide residues, coupled with observations of changes in water quality parameters, fungal diversity, and the distribution of collected snails and plants.

## Materials and Methods

The study was conducted through four successive seasons from January to December 2021, in eight agricultural water courses at Giza Governorate and Tanta (Gharbeya Governorate). The sites and their coordinates are shown in Table 1.

**Table 1:** Coordinates of the investigated sites

	No	Site	Coordinates
Giza	1	El-Zumareya Canal, Oseem	30°05′55.3" N 31°08′59.1"E
	2	Tabeq Canal, Oseem	30°06′45.0" N 31°08′14.0"E
	3	El-Mansoureya Canal, Ezbet Abdel-Samad	30°06′48.0" N 31°04′34.5"E
	4	El-Prinsessa Canal, Ezbet El-Bashary	30°03′33.1" N 31°04′59.8"E
Tanta	5	Ezbet Matar	30°47′29.7" N 31°02′02.8"E
	6	Kafr Krtam	30°47′12.5" N 31°02′38.5"E
	7	Meet Hebash village	30°45′51.6" N 31°06′36.6"E
	8	Kafr Abou Dawood	30°45′45.7" N 31°01′09.1"E

### Sampling

All sampling (including physical–chemical parameters, pesticides, plants, fungi, and snails) was conducted on the same days for each site. Pesticide samples were prepared for analysis, while fungi samples were cultured within 12 h.

### Determination of Physico-Chemical Parameters

A portable pH meter [Hanna Instruments (HI) 9024, USA] was used to measure water temperature and hydrogen ion concentration (pH). Electrical conductivity (EC) and total dissolved solids (TDS) were measured using a portable conductivity meter (HI 9635, USA). Dissolved oxygen was measured using a portable DO meter [Hanna Instruments (HI) 98,193, USA]. All parameters were measured in situ at midday 20 cm below the water surface (Jannat et al. 2019) and recorded in the field survey sheets using APHA (1998) procedures.

### Pesticides Analysis and Extraction

From each site, one liter of water samples was seasonally collected in a dark bottle at the water surface and another at 40 cm below the water surface (Dahshan et al. 2016) and then transferred to the laboratory, mixed well, filtered, and prepared for analysis. Sample preparation involved isolating the target analysts from the sample matrix. Following extraction, a clean-up phase was conducted to remove any interfering substances that could compromise the integrity of the analysis. Finally, the process culminated in the meticulous preparation of a sample specifically tailored for chromatography, ensuring optimal conditions for separation and detection. Executing these steps with precision enhances the overall efficacy of the analytical method employed (Barceló and Hennion 1997). The QuEChERS features straightforward steps that are fast and do not require specialized equipment, allowing for high recovery rates of various pesticides from fruits, vegetables, and other food products using a 1:1 sample-to-solvent ratio. For water samples, a 4:1 ratio is recommended, avoiding PSA clean-up (Abdel Ghani and Hanafi 2016). All standard chemicals used for the pesticide extraction were purchased from Sigma–Aldrich™ (Germany). MgSO_4_ (anhydrous) and NaCl were brought from Merck™, Germany, while the stock solution and working solutions were obtained from BDH, UK.

According to the QuEChERS method for sample preparation, a 4:1 ratio was used for water samples. Ten ml of water sample with 2.5 ml acetonitrile (1% acetic acid) was put in a 15 ml tube, then capped, and kept in the freezer at -18 °C for 15 min before adding 4 g MgSO_4_ and one gram NaCl. The tubes were hand-shaken vigorously for 1 min and centrifuged under cooling conditions (5 °C) for 5 min at 4400 rpm. After that, one ml of the supernatant layer was taken to a 2-ml vial containing 500 mg MgSO_4_. The vials were shaken and then centrifuged under cooling conditions for 2 min at 4400 rpm, and then, the extract was transferred to a PTFE-capped vial from Shimadzu™, Japan, used for GC–MS analysis.

The determination of pesticide residues was performed by gas chromatography with mass spectrometric detection using a Shimadzu GC/MS-Q2010 Ultra (Shimadzu Corporation™, Japan), which is equipped with Shimadzu AOC-20i autosampler™ and a split/splitless injector in the split mode at 260 °C. Chromatographic separations were conducted using a RXISIL 5 MS fused silica column sourced from Restek™, USA. The oven temperature was 200 °C for 2 min, raised to 290 °C at 15 min, and then held for 4 min. The injected volume was one μL (Abdel Ghani and Hanafi 2016). The MS detector was run in scan mode from 50 to 500 m/z, and after that, SIM mode was operated. The ion source was electron ionization (EI) mode, the ion source temperature was 300 °C, the quadrupole temperature was 180 °C, the transfer line temperature was 280 °C, and the electron energy was 70 eV. Three ions were selected for each pesticide. The highest relative abundance ion was used as the quantifier ion, while the other ions were used for confirmation as qualifier ions (*see the supplementary materials*). The recovery mean was determined in five replicates at fortification level 0.01 mg by spiking ten ml of distilled water with the standard solution, all done by a single analyst in a day. The validation of the analytical method was according to SANTE/12682/2019 guidance, which included assessing accuracy, LOD, LOQ, precision, linearity, and trueness (bias). The value of limit of detection (LOD) and the limit of quantification (LOQ) were 3.3 and 10 μg/L, respectively, for the detected pyrethroid and triazole residues in water (*see the supplementary materials*).

### Fungal Isolation and Purification

Fungi were isolated from the collected water samples following the method of Kaufmann et al. (1988). Water was collected in sterilized plastic falcon tubes (50 ml) below 20 cm of the water surface. The falcon tubes were filled with water, then immediately capped, kept in an ice box, and transported to the laboratory for analysis within 12 h.

From each water sample, 1 ml was diffused in a test tube containing 9 ml of sterile distilled water. Serial decimal dilutions were prepared from the original water sample concentrations and pipetted onto Petri dishes containing Czapek agar (Madrid, Spain), malt extract, and Sabouraud dextrose agar (Heywood, BL9 7JJ, UK) added to 500 mg/L of chloramphenicol (Himachal Pradesh, India). For each concentration, three replicate plates were prepared and incubated at (28 °C ± 2 °C) for a week, and then, the grown fungal species were purified by streaking repeatedly on the same medium (Anon 1996). Isolated species were sent for molecular confirmation.

### Molecular Identification of Isolated Fungal Strains

DNA extraction, PCR, and sequencing (18SrRNA) were used for molecular identification. The isolated fungi were identified using the nuclear ribosomal internal transcribed spacer (ITS) region 1, 2, with the short structural gene (5.8 S). The ITS region was chosen to identify fungi due to their recognition as prevalent markers in this field (Schoch et al. 2012; El-Hady et al. 2014). To purify the PCR products and eliminate excess PCR primers and dNTPs, a Montage PCR Clean-up kit (Millipore) was used. Sequencing was conducted using the Big Dye Terminator cycle sequencing kit (Applied Biosystems, USA). The determination of sequencing products was performed using the Applied Biosystems model 3730XL automated DNA sequencing system (Applied Biosystems, USA). In the current study, the primers used for identification were ITS1 (5'- TCC GTA GGT GAA CCT GCG G -3') and ITS4 (5'- TCC TCC GCT TAT TGA TAT GC -3').

### Snail Samples

Snail sampling was seasonally performed using a standard flat wire mesh scoop with a mesh size of 2 cm Takougang et al. (2008). From each site, snails were collected and placed in a numbered plastic aquaria containing some water, transferred to the laboratory, identified according to Ibrahim et al. (1999), and counted and examined for natural trematode infection (El-khayat et al. 2013).

### Aquatic Plants

Plants coexisting with snails at the sampling sites were observed in situ and recorded in the field sheets. A sample from each plant was put in a plastic bag, labeled, transferred to the lab, and identified to Zahran and Willis (2009). The frequency percentage of each plant was determined by dividing the number of sites where it heavily appeared by the total number of sites (Nunes et al. 2019).

### Statistical Analysis

All physical parameters were expressed as mean and standard deviation and compared between Giza and Tanta using a t-test at significant levels of *p* < *0.05, p* < *0.01, and p* < *0.001*. Seasonal variation in pesticides restudies was performed using one-way ANOVA test at *p* < *0.05*. Pearson's correlation coefficient was used to state the relationship between the presence of pesticides and the distribution of snail species at *p* < *0.05* and *p* < *0.01* using the SPSS software package, version 20. Principal component analysis (PCA) was done to minimize large data and analyze the relationships among the observed variables using Minitab software, version 14. Conical corresponding analysis (CCA) was performed to show the distribution of different snail species according to water physical parameters and aquatic plants using Past software, version 4.03.

## Results

### Physicochemical Parameters of Watercourses

**Fig. 1:**
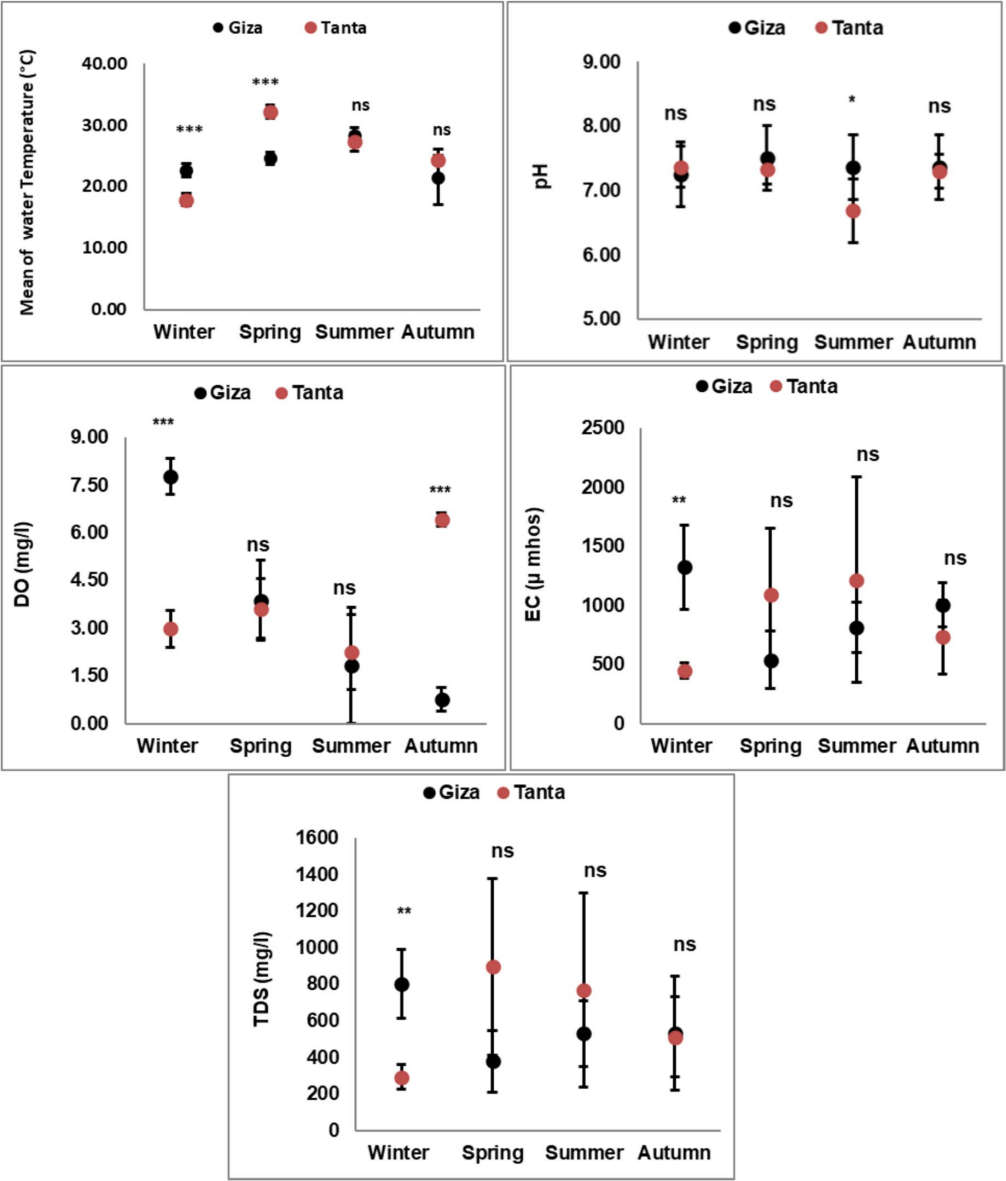
Physical parameters of water samples collected from Giza Governorate and Tanta, during four seasons (during 2021) *,**, and *** refer to significant results at *p* < *0.05, p* < *0.01*, and *p* < *0.001*, respectively, using t-test

Data in Fig. 1 are summarized the seasonal variations in water physical parameters between the two investigated governorates. There were significant differences in water temperature of Giza Governorate and Tanta during the winter and spring seasons. The highest water temperature was recorded (32.23 ± 1.11 °C) in spring, while the lowest was in winter (17.88 ± 1.03 °C) at Tanta. On the other hand, there were no significant differences in temperature values at the two governorates during summer and autumn. pH levels in the two governorates tend to be neutral during all seasons, except for the summer season in Tanta, where pH was slightly decreased to 6.69 ± 0.49. Dissolved oxygen (DO) values showed very highly significant differences between the two governorates during winter and autumn, where the highest value of DO was (7.77 ± 0.57 mg/l) during winter, while the lowest was (0.75 ± 0.21 mg/l) during autumn in Giza. No significant results were observed in electrical conductivity (EC) between the two governorates in all seasons, except for the winter season, where the highest EC was (1326 ± 357.9 µ mhos) in Giza, while the lowest was (448.5 ± 62.9 µ mhos) in Tanta. In the same manner, total dissolved solids (TDS) values showed significant differences in winter, where the highest value was (801.75 ± 188.3 mg/l) in Giza and the lowest was (293.5 ± 68.03 mg/l) in Tanta.

### Concentrations of Pyrethroid and Triazole Pesticides

As shown in Table 2, the results of the analysis of pyrethroids insecticides in water samples collected in Giza Governorate indicated that the concentration of deltamethrin (32.69 µg/L) and Es-fenvalerate (39.18 µg/L) was the highest recording in spring and summer, respectively. Meanwhile, lambada-cyhalothrin (29.27 µg/L) and permethrin (32.76 µg/L) were the highest in autumn (Table 2).

**Table 2:** Seasonal variation in pyrethroid and triazole pesticides concentrations (µg/l) in water samples of Giza Governorate during four seasons (during 2021)

Pesticicdes	Code	Winter	Spring	Summer	Autumn	*F–value*	*P–value*	
Pyrethroid	Bifenthrin	Bif	1.31^a^ ± 0.59	6.512^b^ ± 1.17	1.836^a^ ± 0.93	14.75^c^ ± 2.06	330.8	<0.01
	Cypermithrin	Cyp	–	3.64 ± 0.38	–	2.86 ± 0.51	–	–
	Deltamethrin	Delt	13.39^a^ ± 4.49	9.64^ac^ ± 2.64	39.18^b^ ± 7.35	5.73^c^ ± 0.81	204.2	<0.01
	Es-fenvalerate	Es-fen	11.89^a^ ± 1.38	32.69^b^± 4.88	11.69^a^ ± 2.79	9.28^a^± 1.15	135.9	<0.01
	Fenpropathrin	Fen	0.73^ab^ ± 0.28	0.40^a^± 0.01	1.33^b^ ± 0.72	3. 82^c^ ± 0.86	64.25	<0.01
	Lambada-cyhalothrin	Lam-cy	6.69^a^ ± 1.76	23.11^b^ ± 3.13	5.23^a^ ± 3.09	29.27^c^ ± 3.84	205.85	<0.01
	Permethrin	Per	2.64^a^ ± 0.56	13.47^b^ ± 1.08	17.72^c^ ± 5.62	32.76^d^ ± 6.35	146.34	<0.01
Triazole	Difenoconazole	Dif	6.54^a^ ± 1.79	42.95^b^ ± 5.45	413.34^c^ ± 97.43	–	2924.18	<0.001
	Diniconazole	Dini	–	0.60 ± 0.08	–	0.51 ± 0.03	–	–
	Epoxiconazole	Epox	–	–	–	5.02 ± 0.67	–	–
	Flusilazole	Flus	1.49^a^ ± 0.67	0.19^a^ ± 0.02	16.33^b^ ± 3.25	0.35^a^ ± 0.03	391.217	<0.001
	Penconazole	Penc	–	–	5.52 ± 0.98	1.61 ± 0.66	–	–
	Propiconazole	Prop	0.32^a^ ± 0.01	0.82^b^ ± 0.05	2.98^c^ ± 0.03	1.44^d^ ± 0.04	867.99	<0.001
	Tebuconazole	Tebu	–	521.57^a^ ± 60.61	224.38^b^ ± 40.82	8.48^c^ ± 1.03	2831.03	<0.001
	Tetraconazole	Tetr	60.08^a^ ± 5.84	543.18^b^ ± 34.27	4.87^c^ ± 0.09	89.45^d^ ± 5.26	12894.8	<0.001

Different letters refer to significant results at *p* < *0.05,* while the same letters refer to insignificant results at *p* > *0.05* using one-way ANOVA

In Tanta, pyrethroid pesticides: Deltamethrin (25.85 µg/L) and Es-fenvalerate (30.72 µg/L) were the highest recording in spring and summer, respectively (Table 3).

**Table 3:** Seasonal variation in pyrethroid and triazole pesticides concentrations (µg/l) in water samples of Tanta during four seasons (during 2021)

	Pesticicdes	Code	Winter	Spring	Summer	Autumn	*F– value*	*P – value*
Pyrethroids	Bifenthrin	Bif	0.55^a^ ± 0.06	0.90^b^ ± 0.06	0.04^c^± 0.01	1.53^d^ ± 0.67	661.228	<0.001
	Cypermithrin	Cyp	2.61^a^ ± 0.56	0.92^b^ ± 0.04	0.29^c^ ± 0.03	0.85^b^ ± 0.03	137.021	<0.001
	Deltamethrin	Delt	12.39 ±1.17	25.85 ± 3.11	11.11 ± 2.21	2.85 ± 0.22	3220.237	<0.001
	Es-fenvalerate	Es-fen	15.08^a^ ± 2.65	0.82^b^ ± 0.03	30.72^c^ ± 5.39	4.90^d^ ± 0.81	13123.72	<0.001
	Fenpropathrin	Fen	0.18^a^ ± 0.23	–	0.54^b^ ± 0.05	0.64^c^ ± 0.01	117.554	0.001
	Lambada-cyhalothrin	Lam-cy	11.20^a^ ± 3.69	5.32^b^ ± 0.91	3.19^c^ ± 0.84	2.42^c^ ± 0.28	471.342	<0.001
	Permethrin	Per	3.31^a^± 0.45	5.52^b^ ± 1.85	17.53^c^ ± 4.37	10.77^d^ ± 1.68	976.417	<0.001
Triazoles	Difenoconazole	Dif	20.36 ± 2.19	19.03 ± 2.83	23.34 ± 5.45	–	6.58	0.083
	Diniconazole	Dini	0.95 ± 0.02	–	–	0.76 ± 0.05	–	–
	Epoxiconazole	Epox	23.3 ± 4.98	–	–	2.24 ± 0.46	–	–
	Flusilazole	Flus	–	0.76^a^ ± 0.04	8.53^b^ ± 1.98	1.67^c^ ± 0.09	1170.32	<0.001
	Penconazole	Penc	2.13^a^ ± 0.07	3.28^b^ ± 0.67	8.34^c^ ± 0.75	0.39^d^ ± 0.01	891.56	<0.001
	Propiconazole	Prop	0.03^a^ ± 0.01	0.47^b^ ± 0.05	0.02^a^ ± 0.01	0.26^d^ ± 0.14	297.10	<0.001
	Tebuconazole	Tebu	129.39^a^ ± 19.8	4.70^b^ ± 1.50	83.67^c^ ±6.61	2.09^b^ ± 0.58	3506.85	<0.001
	Tetraconazole	Tetr	50.75^a^ ± 9.75	97.69^b^ ± 9.34	–	69.82^c^ ± 9.02	442.067	<0.001

Different letters refer to significant results at *p* < *0.05,* while the same letters refer to insignificant results at *p* > *0.05* using one-way ANOVA

The results of the analysis of triazole fungicides in Giza indicated that the concentrations of tebuconazole (521.57 µg/L) and tetraconazole (543.18 µg/L) were the highest in spring and difenoconazole (413.34 µg/L) recorded its highest value in summer, as shown in Table 2.

In Tanta, the triazole pesticides: Tebuconazole was the highest in winter (129.39 µg/L) and summer (83.67 µg/L), while tetraconazole was the highest in spring (97.69 µg/L) and autumn (69.82 µg/L) (Table 3).

### Frequency of Fungal Species

Concerning the frequency of the fungal species, Table 4 shows the key and the accession numbers of 21 molecularly identified fungal species related to four fungal genera, *Aspergillus*, *Fusarium, Penicillium*, and *Trichoderma,* isolated from water samples, representing Giza Governorate and Tanta (Gharbeya Governorate).

**Table 4:** Key and accession numbers of fungal species isolated from water samples representing Giza Governorate and Tanta

Key	Fungal species with accession number
Asp.1	*Aspergillus brevipes OQ642272*
Asp.2	*Aspergillus cejpii OM802834*
Asp.3	*Aspergillus flavus OM802831*
Asp.4	*Aspergillus flavus OM802833*
Asp.5	*Aspergillus fumigatus OM722121*
Asp.6	*Aspergillus fumigatus OM802836*
Asp.7	*Aspergillus fumigatus OM807137*
Asp.8	*Aspergillus niger OM802854*
Asp.9	*Aspergillus quadrilineatusOM455463*
Fus.1	*Fusarium andiyazi OM758342*
Fus.2	*Fusarium oxysporum OM832837*
Fus.3	*Fusarium oxysporum OM802851*
Fus.4	*Fusarium oxysporum f. sp. cucumerinum OM722087*
Pen.1	*Penicillium sp. OQ658672*
Pen.2	*Penicillium commune OM802848*
Pen.3	*Penicillium griseofulvum OM722119*
Pen.4	*Penicillium polonicum OM758307*
Tal.1	*Talaromyces stipitatus OM802855*
Tri.1	*Trichoderma asperellum OM758334*
Tri.2	*Trichoderma capillare OM802849*
Wes.1	*Westerdy kelladispersa OM832838*

Generally, *Aspergillus* sp. was the most prevalent among all fungal genera followed by *Pencillium* sp., then *Fusarium*, and *Trichoderma*. The largest diversity of fungi was observed during spring in Tanta and autumn in Giza.

**Fig. 2:**
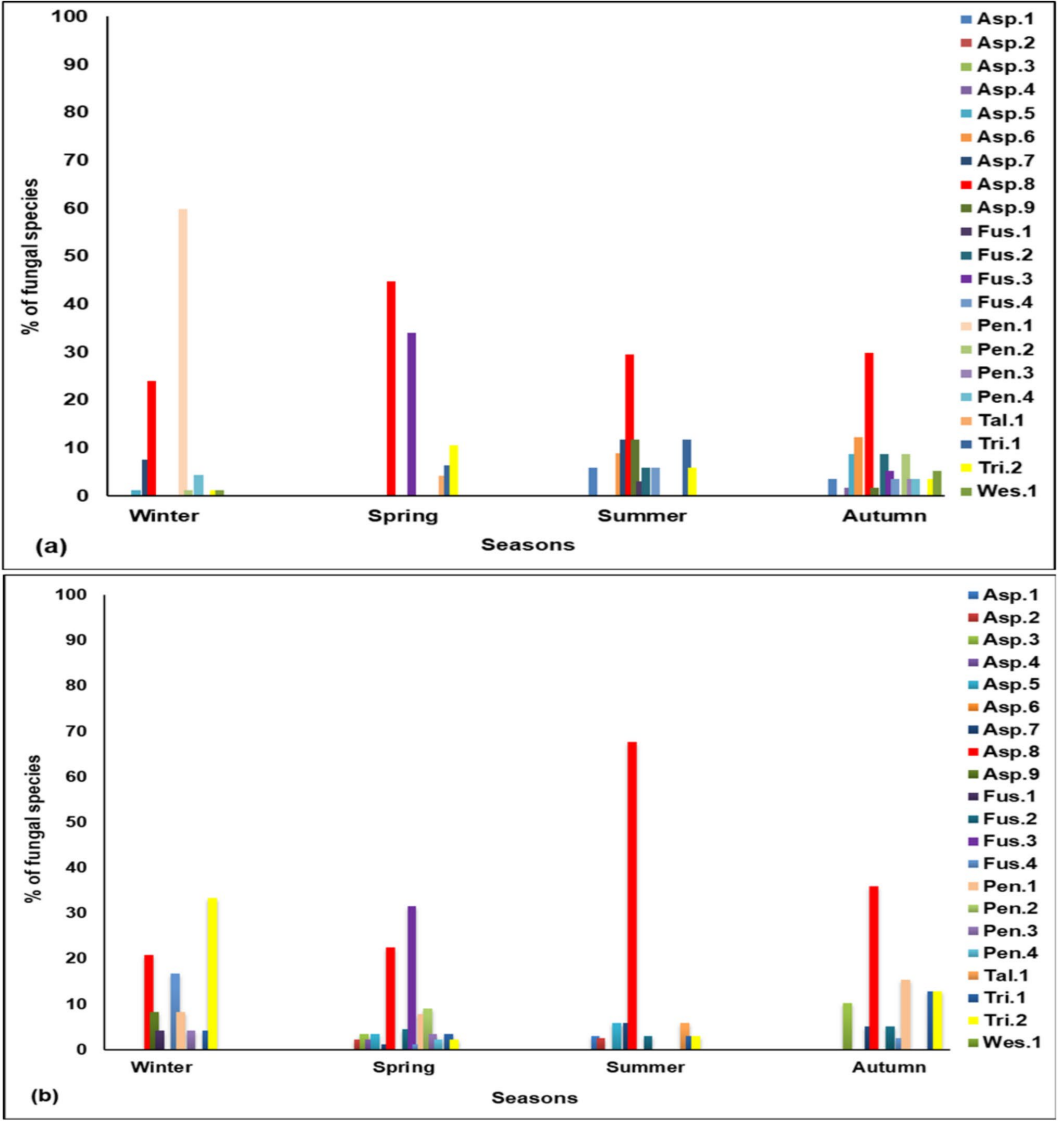
Seasonal variation in fungal frequency in water courses representing Giza Governorate **a** and Tanta **b**

Data illustrated in Fig. 2a indicated that the distribution and percentages of fungal species in Giza Governorate depend on seasons of collection. The fungus *Penicillum* sp. (Pen. 1) recorded the highest percentage of fungal species (59.78%) during winter, followed by *A. niger* (Asp.8) (44.69%) during spring, which was the most abundant among fungal species approximately at all seasons. During spring, *F. oxysporum* (Fus.3) recorded (34.04%), while *P. commune* (Pen. 2)*, T.capillare* (Tri.2), and *Westerdy kelladispersa* (Wes.1) showed the same lowest percentage (1.09%) among all fungal species during spring (Fig. 2a).

In Tanta (Fig. 2b), the results indicated that *A. niger* (Asp.8) was the most frequent in all seasons and exhibits the highest percentage during summer (67.65%), followed by *T. capillare* (Tri.2) during winter (33.33%) and *F. oxysporum* (Fus.3) during spring (31.47%). Meanwhile, the same lowest percentages were (1.12%) for *A. fumigatus* (Asp.7) and *F. oxysporum f.* sp*. Cucumerinum* (Fus.4), and (2.25%) for *A. cejpii* (Asp.2), *A. flavus* (Asp.4), and *P. polonicum* (Pen.4) during spring in Tanta (Fig. 2b).

### Abundance of Snail Species

**Fig. 3:**
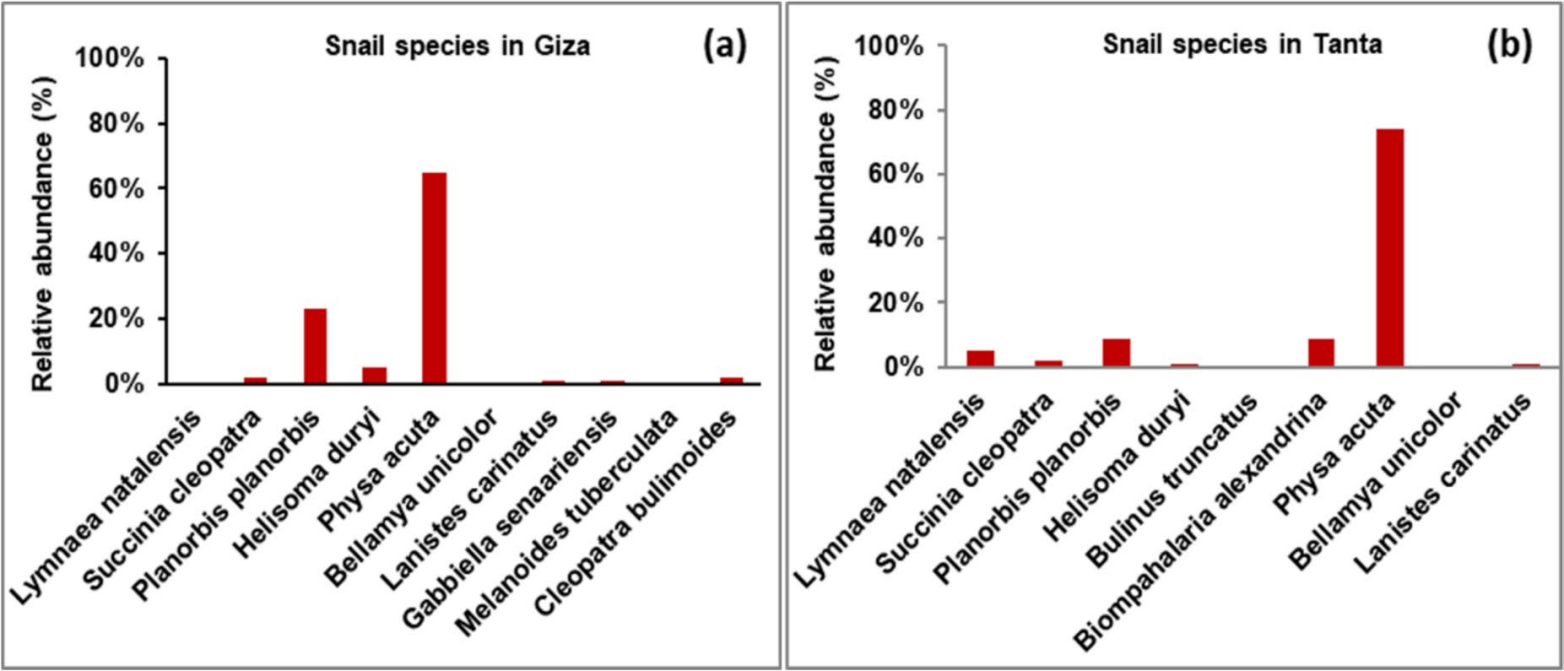
Relative abundance of snail species in Giza Governorate **a** and Tanta **b** during four seasons (during 2021)

Results of snail diversity recorded 10 and 9 species in Giza and Tanta, respectively (Fig. 3a and b). These species belonged to Pulmonate snails (*Lymnaea natalensis*, *Succinia cleopatra*, *Planorbis planorbis*, *Helisoma duryi*, *Bulinus truncatus, Biomphalaria alexandrina*, and *Physa acuta*) and Prosobranch snails (*Bellamya unicolor*, *Lanistes carinatus*, *Gabbiella senaariensis*, *Melanoides tuberculata,* and *Cleopatra bulimoides*). *P. acuta* and *P. planorbis* were the highest abundant (65 and 23%, respectively) in Giza, while *P. acuta* was the highest (74%) in Tanta.

*L. natalensis*, *B. unicolor*, and *M. tuberculate* were the lowest abundant (0.3%) in Giza, while *B. unicolor* and *B. truncates* were species in Tanta (0.1%).

### Relative Abundance of Aquatic Plants

**Fig. 4:**
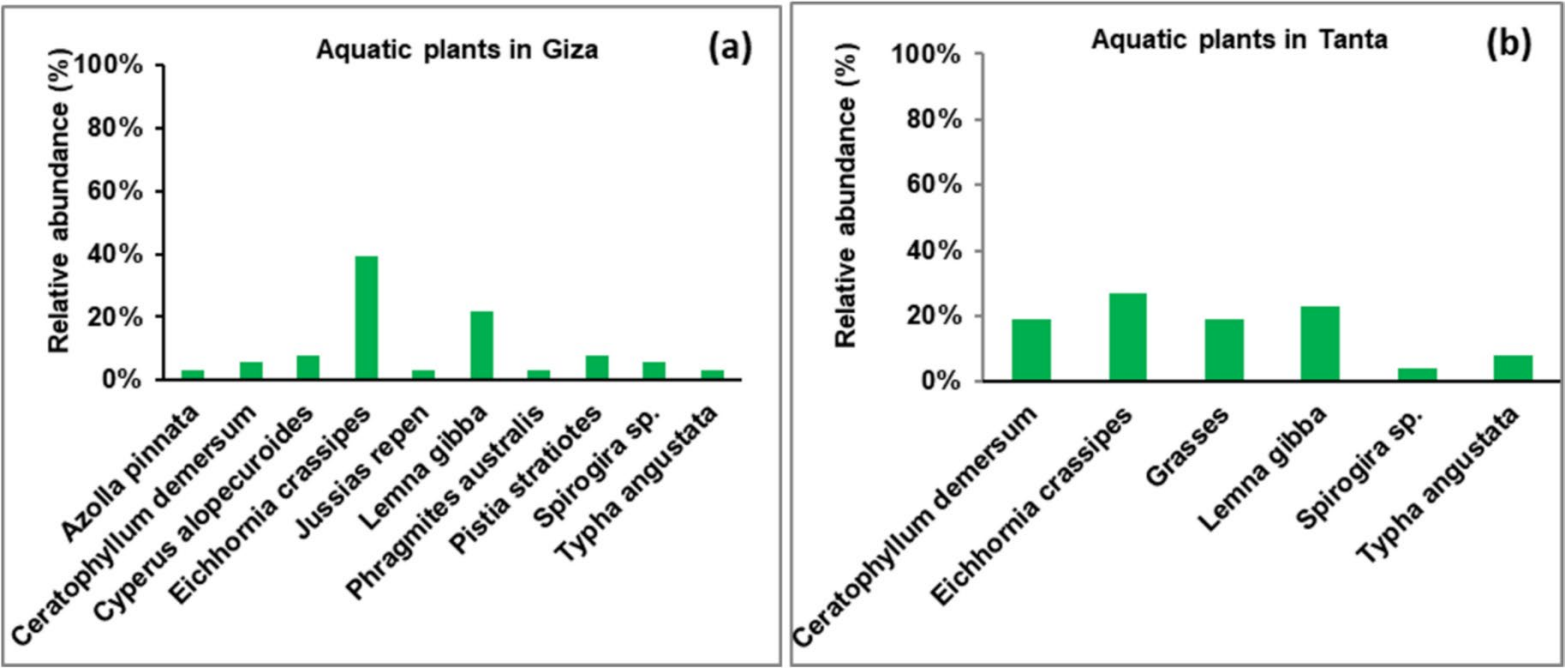
Relative abundance of aquatic plants in Giza Governorate **a** and Tanta **b** during four seasons (during 2021)

Regarding the aquatic plant diversity in the two investigated governorates, results showed that ten species were detected in Giza, while six species were in Tanta. Besides, *Eichhornia crassipes* and *Lemna gibba* were the dominant species in the two governorates, with the relative abundance of the two plants (39 and 22%, Fig. 4a) in Giza and (27 and 23%, Fig. 4b) in Tanta, respectively. The lowest abundance (3%) was recorded for *Azolla pinnata*, *Jussias repen*, *Phragmites australis,* and *Typha angustata* in Giza, while *Spirogira* sp. recorded the lowest abundance (4%) in Tanta.

### Correlation Between Variables

**Fig. 5:**
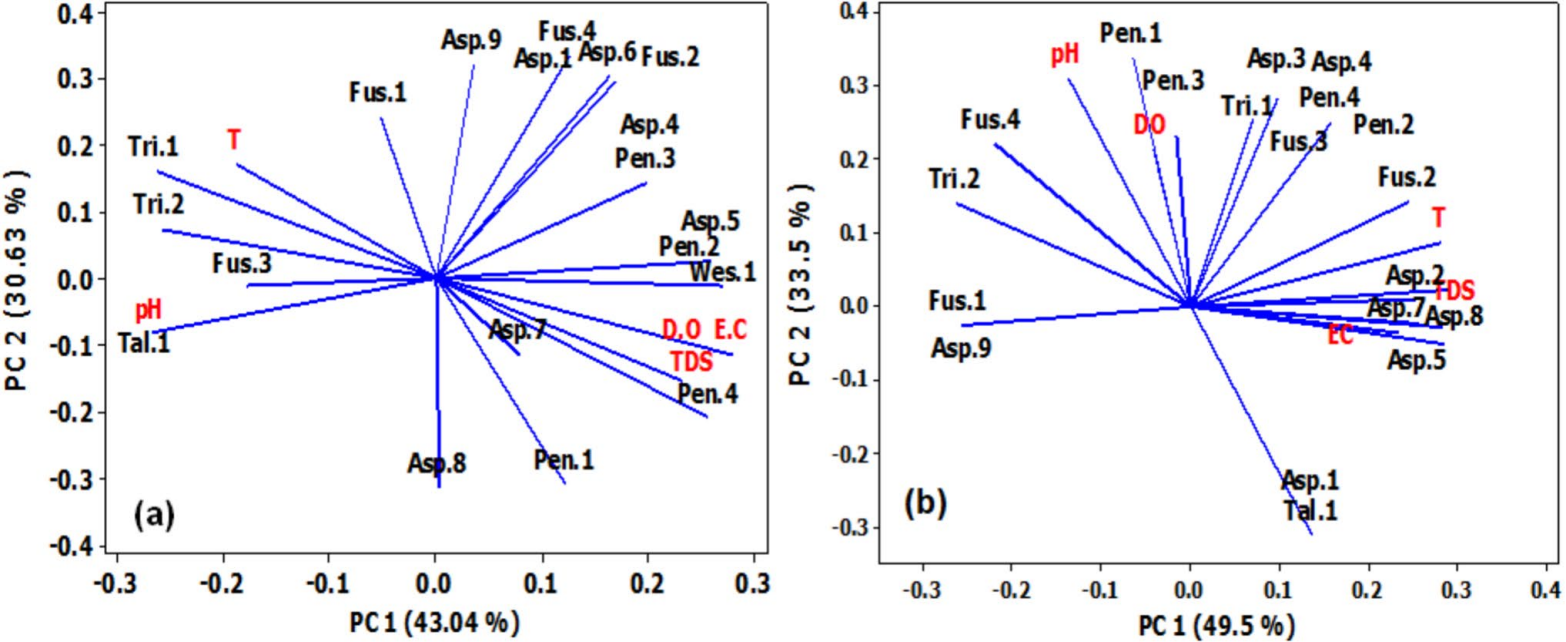
Principal component (PC) analysis showing the correlation patterns between fungi species and physical parameters in Giza Governorate **a** and Tanta **b**

In Giza, the first two principal components (PC) explained 73.67% of the variation in the data of fungi species and physical parameters (Fig. 5a). Temperature was positively correlated with fungi species (Tri 1, Tri 2, Fus. 3), and pH was positively correlated with (Tal.1). Meanwhile, EC, TDS, and DO showed positive correlations with Asp.5, Pen. 2, Pen. 4, and Wes.1.

Regarding the association between fungi species and physical parameters in Tanta (Fig. 5b), data showed that the first two PC represented 80 % of the variation in the data. The 1st PC1 contained temperature, TDS, EC which were positively correlated with fungi species (Asp.2, Asp.5, Asp.7, and Fus.2), while they showed negative correlations with fungi species (Asp.9 and Fus.1). On the other hand, the 2nd PC2 contained pH and DO which showed strong positive correlations with fungi species Pen.1 and Pen. 3, respectively, while they displayed negative correlations with fungi species (Asp.1 and Tal.1).

**Fig. 6:**
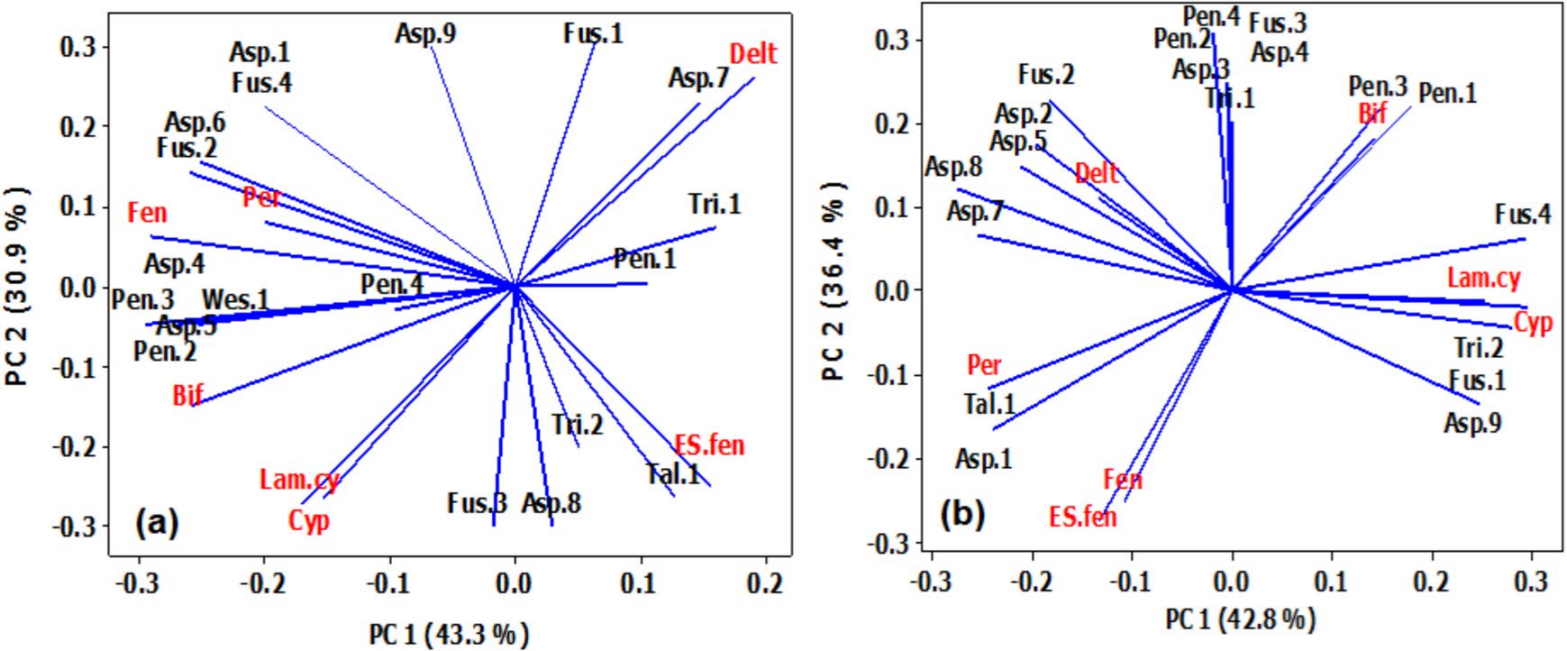
Principal component (PC) analysis showing the correlation patterns between fungi species and pyrethroid concentrations in Giza Governorate **a** and Tanta **b**

Regarding the association between fungi species and pyrethroid concentrations in Giza (Fig. 6a), PC indicated that 74.3% of the variation in the data. The 1st PC1 contained bifenthrin and fenpropathrin which were positively correlated with fungi species (Asp.4, Asp. 5, Pen.2, and Pen.3). The 2nd PC2 contained deltamethrin which showed strong positive correlation with fungi species (Asp.7) and permethrin was positively correlated with Asp.6 and Fus.2, while cypermithrin and lambada-cyhalothrin were negatively associated with (Asp.7).

In Tanta, the first two PC represented 79.2% of the variation among fungi species and pyrethroid concentrations (Fig. 6b), and the 1st PC1 showed a strong negative correlation between pyrethroids (cypermithrin and lambada-cyhalothrin) and fungi species (Asp.7 and Asp.8), while they showed positive correlations with fungi species (Asp.9, Fus.1, Fus.4, and Tri.2). On the other hand, the 2nd PC2 exhibited a strong negative association between Es-fenvalerate and fenpropathrin and fungi species (Asp.3, Asp.4, Fus.3, Pen.2, Pen.4, and Tri.1).

**Fig. 7:**
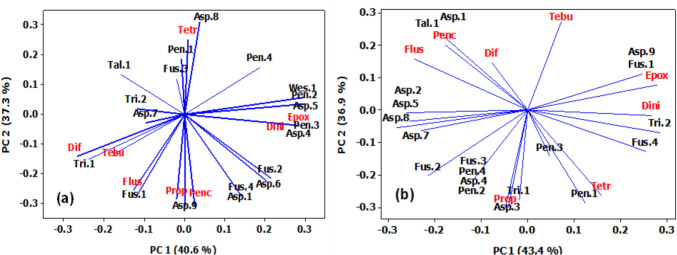
Principal component (PC) analysis showing the correlation patterns between fungi species and triazole concentrations in Giza Governorate **a** and Tanta **b**

Data in Fig. 7a displayed 77.9% of the variation in the data of fungi species and triazole concentrations in Giza. The 1st PC1 contained diniconazole (Dini) and epoxiconazole (Epox) which showed strong positive correlations with fungi species (Asp.4, Asp.5, Pen.2, Pen.3, and Wes.1). Meanwhile, the 2nd PC2 contained penconazole and propiconazole which showed strong positive correlations with fungi species (Asp.1, Asp.9, Fus.1, and Fus.4). On the other hand, the first two PC explained 80.3% of the variation in the data between fungi species and triazole concentrations in Tanta (Fig. 7b), the 1st PC1 contained diniconazole (Dini) and epoxiconazole (Epox) which showed strong positive correlations with fungi species (Asp.9, Fus.1, Fus.4, and Tri.2), while the 2nd PC 2 contained propiconazole (Prop) and tetraconazole (Tetr), which were positively correlated with fungi species (Asp. 3, Tri. 1, and Pen.1).

**Fig. 8:**
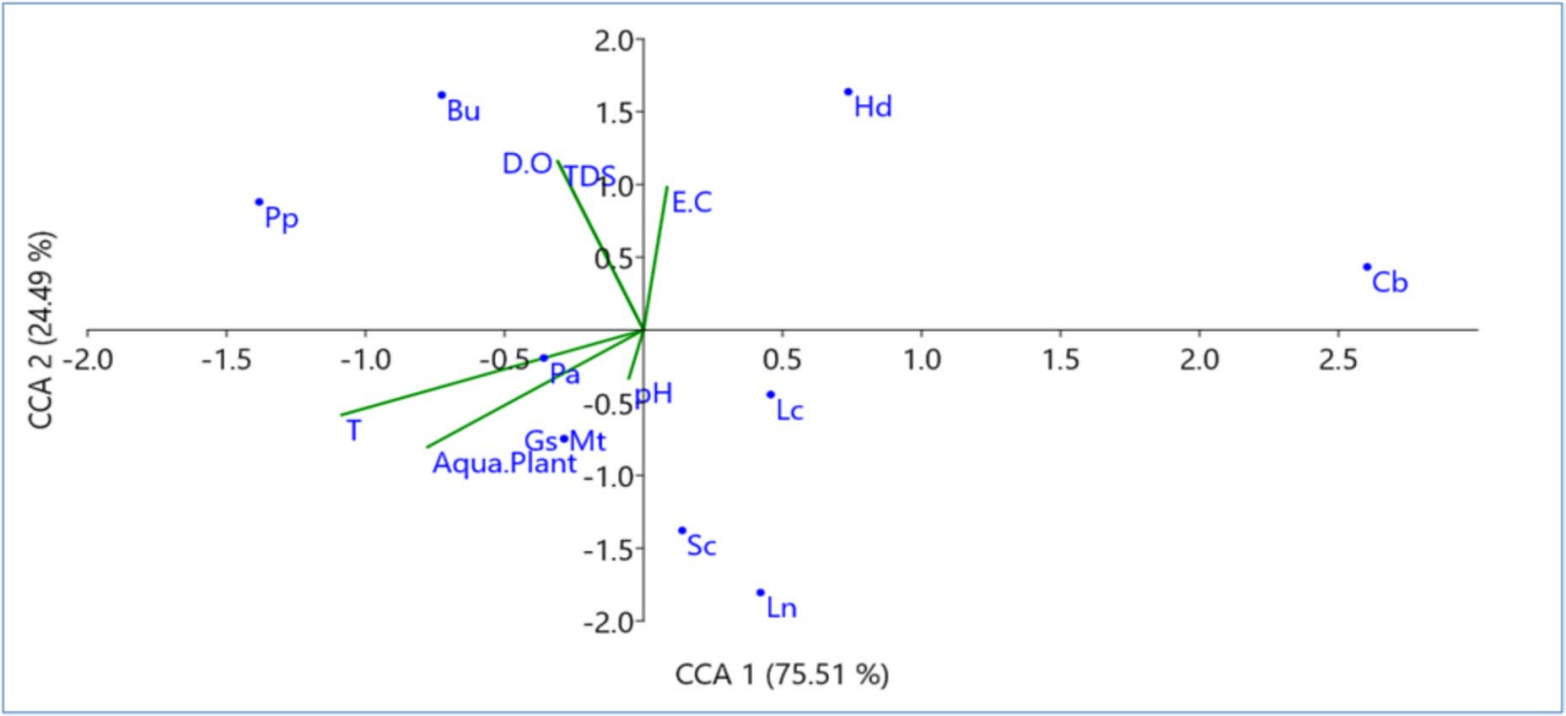
Conical corresponding analysis (CCA) showed distribution of different snail species from Giza Governorate according to water physical parameters and aquatic plants

**Fig. 9:**
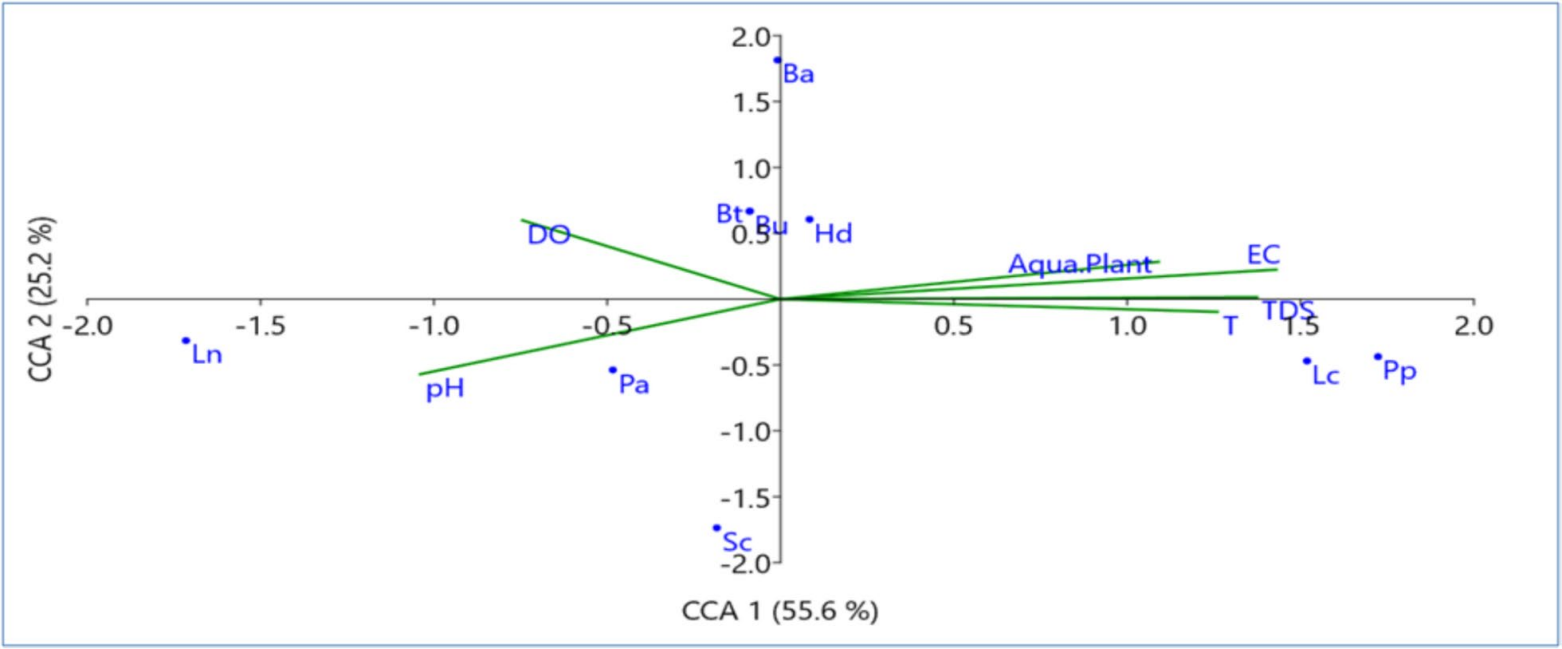
Conical corresponding analysis (CCA) showed distribution of different snail species from Tanta according to water physical parameters and aquatic plants

Concerning the distribution of different snail species according to water physical parameters and aquatic plants, in Giza, conical corresponding analysis (CCA) showed that distribution of *H. duryi*, *L. carinatus*, *C. bulimoides*, *L. natalensis*, and *S. cleopatra* was negatively correlated with temperature, D.O, pH, TDS, while *B. unicolor* and *H. duryi* showed strong associations with TDS and D.O., and EC. Aquatic plants have positive impact on *G. senaariensis* and *M. tuberculata* distribution, and water temperature (T). *P. acuta* was positively distributed with pH as shown in Fig. 8. In Tanta, CCA showed positive effect of temperature, EC, TDS, and aquatic plants on the distribution of *L. carinatus*, *P. planorbis*, *H. duryi* snail species, while *P. acuta* and *L. natalensis* were positively correlated with pH. Meanwhile, D.O has a positive effect on the distribution of *B. truncates* and *B. unicolor* as shown in Fig. 9.

Regarding the impact of both pyrethroid and triazole concentrations on the distribution of snails, the results of correlations are presented in Tables 5 and 6, respectively. In Giza, *B. unicolor* was negatively correlated with bifenthrin; *G. senaariensis* and *C. bulimoides* were positively correlated with Fenpropathrin. *S. cleopatra* and *P. acuta* showed strong positive and negative correlations with permethrin, respectively (Table 5). *P. planorbis* was negatively correlated with propiconazole, while *G. senaariensis* was positively correlated with flusilazole. Meanwhile, *M. tuberculata* and *C. bulimoides* showed positive correlations with diniconazole and epoxiconazole (Table 5).

**Table 5:** Correlation analysis between pyrethroid and triazole pesticides concentrations and different species of snails collected from Giza Governorate

Pesticides		*Ln*	*Sc*	*Pp*	*Hd*	*Pa*	*Bu*	*Lc*	*Gs*	*Mt*	*Cb*
Pyrethroid	Bifenthrin	0.711	0.827	−0.335	0.244	−0.712	−0.992^**^	0.595	−0.432	0.913	0.868
	Cypermithrin	0.374	0.556	−0.107	0.043	−0.449	−0.875	0.189	−0.577	0.603	0.513
	Deltamethrin	−0.753	−0.368	−0.264	−0.622	0.188	0.75	−0.118	0.867	−0.738	−0.594
	Es-fenvalerate	−0.581	−0.300	0.275	−0.463	0.293	−0.005	−0.628	−0.289	−0.433	−0.515
	Fenpropathrin	0.883	0.823	−0.464	0.446	−0.741	−0.727	0.87	−0.091	0.949	0.977^*^
	Lambada-cyhalothrin	0.488	0.538	−0.027	0.189	−0.409	−0.881	0.188	−0.666	0.666	0.56
	Permethrin	0.257	0.955^*^	−0.921	−0.382	−0.990^**^	−0.749	0.877	0.371	0.626	0.735
Triazole	Difenconazole	−0.934	−0.343	−0.277	−0.851	0.159	0.618	−0.247	0.777	−0.805	−0.683
	Diniconazole	0.904	0.829	−0.347	0.467	−0.712	−0.881	0.75	−0.333	1.000^**^	0.981^*^
	Epoxiconazole	0.904	0.829	−0.347	0.467	−0.712	−0.881	0.75	−0.333	1.000^**^	0.981^*^
	Flusilazole	−0.505	0.000	−0.576	−0.594	−0.177	0.484	0.281	0.982^*^	−0.414	−0.231
	Penconazole	−0.042	0.592	−0.918	−0.474	−0.718	−0.119	0.789	0.864	0.186	0.376
	Propiconazole	−0.210	0.69	−0.974^*^	−0.741	−0.815	−0.347	0.692	0.744	0.173	0.336
	Tebuconazole	−0.664	0.23	−0.537	−0.942	−0.349	−0.149	0.034	0.446	−0.284	−0.205
	Tetraconazole	0.104	−0.137	0.550	0.223	0.264	−0.36	−0.496	−0.856	0.106	−0.067

(*, **) symbols refer to significant difference at *p* < *0.05* and *p* < *0.01*, respectively. *Ln:L. natalensis, Sc: S. cleopatra, Pp: P. planorbis, Hd: H. duryi, Pa: P. acuta, Bu: B. unicolor, Lc: L. carinatus, Gs: G. senaariensis, Mt: M. tuberculata, Cb: C. bulimoides*

**Table 6:** Correlation analysis between pyrithriod and triazole pesticides concentrations and different species of snails collected from Tanta

Pesticides		*L n*	*S c*	*P p*	*H d*	*B t*	*B a*	*P a*	*Bu*	*Lc*
Pyrethroid	Bifenthrin	0.569	−0.348	−0.167	0.187	−0.098	−0.131	−0.122	0.683	−0.916
	Cypermithrin	0.967*	0.564	−0.617	0.651	0.889	−0.961*	0.816	−0.081	−0.792
	Deltamethrin	−0.531	0.541	0.823	0.389	−0.203	0.114	0.215	−0.851	0.881
	Es-fenvalerate	−0.503	0.007	−0.189	−0.543	0.098	0.233	−0.105	−0.293	0.623
	Fenpropathrin	−0.326	−0.672	−0.492	−0.935	−0.174	0.482	−0.597	0.522	−0.132
	Lambada-cyhalothrin	0.778	0.859	−0.363	0.812	0.917	−0.987*	0.984*	−0.494	0.132
	Permethrin	−0.776	−0.795	0.11	−0.954*	−0.7	0.904	−0.898	0.42	0.11
Triazole	Difenconazole	−0.226	0.827	0.424	0.506	0.293	−0.289	0.589	−0.997**	0.95
	Diniconazole	0.796	−0.15	−0.933	−0.055	0.577	−0.506	0.203	0.577	−0.924
	Epoxiconazole	0.855	0.472	−0.829	0.368	0.958*	−0.885	0.734	−0.05	−0.924
	Flusilazole	−.0.971*	−0.347	0.461	−0.656	−0.629	0.808	−0.613	−0.157	0.792
	Penconazole	−0.668	0.425	0.518	0.009	−0.09	0.202	0.117	−0.812	0.998*
	Propiconazole	0.075	−0.493	0.338	0.055	−0.577	0.309	−0.441	0.577	−0.792
	Tebuconazole	0.062	0.673	−0.302	0.17	0.692	−0.482	0.626	−0.692	0.907
	Tetraconazole	0.773	0.067	−0.164	0.56	0.212	−0.484	0.285	0.354	−0.765

(*, **) symbols refer to significant difference at *p* < *0.05* and *p* < *0.01*, respectively. *Ln:L. natalensis, Sc: S. cleopatra, Pp: P. planorbis, Hd: H. duryi, Pa: P. acuta, Bu: B. unicolor, Lc: L. carinatus, Gs: G. senaariensis, Mt: M. tuberculata, Cb: C. bulimoides*

In Tanta, cypermithrin has a strong positive correlation with *L.natalensis* and negative correlation with *B. alexandrina.* On the other hand, lambada-cyhalothrin showed positive correlation with *P. acuta* and negative correlation with *B. alexandrina.* Also, *H. duryi* and *L.natalensis* showed a strong negative correlations with permethrin and flusilazole, respectively, while *B. truncatus* and *L. carinatus* were positively correlated with epoxiconazole and penconazole, respectively. *B. unicolor* showed very strong negative correlation with difenconazole (Table 6).

## Discussion

Water physicochemical parameters play a significant role in aquatic systems (Druzina and Stegu 2007; Ezzat et al. 2012). The maximum water temperatures were 32.23 and 24.63 °C in Tanta and Giza during spring, respectively. These results may be due to climate change and the effect of global warming. Also, El-Khayat et al. (2017) reported that water temperature ranged from 24 to 29 °C during spring at eight Egyptian governorates. Meanwhile, the current pH levels in the two governorates tended to be neutral during all seasons, except summer in Tanta which showed a slight decrease (6.69 ± 0.49). Abdel-Raheem et al. (2018) and Sayed et al. (2021) attributed the slightly acidic pH during summer to anthropogenic activities and increased input of pollutants such as pesticides leading to depression of CO_2_ consumption owing to inhibition of photosynthesis (Usui and Kasubuchi 2011). On the other hand, the highest value of dissolved oxygen (DO) was recorded during winter in Giza, and the lowest was during autumn, and vice versa recorded in Tanta. Saad et al. (2014) observed that the highest DO value was in autumn, while the lowest was in winter. The lowest DO levels reflect a less healthy ecosystem due to the discharge of pollutants and changes in photosynthetic organisms' community. During winter, the highest electrical conductivity (EC) and total dissolved solids (TDS) were in Giza and the lowest was in Tanta. EC and TDS are found to be directly proportional to each other, as EC values reflect the amount of salts dissolved in water (Ghannam et al. 2014; Abdel-Kader et al. 2016; Abdel-Wareth and Sayed 2019), and it may be seasonally changed due to contaminants and human activities.

Concerning the distribution of the fungal species, the molecularly identified fungal species, 21 species related to four fungal genera, *Aspergillus*, *Fusarium, Penicillium*, and *Trichoderma,* were isolated from water samples representing Giza Governorate and Tanta (Gharbeya Governorate). *Aspergillus* sp. was the most prevalent fungal genera followed by *Penicillium* sp. then *Fusarium* and *Trichoderma*. These findings matched with Moharram et al. (1990) and Kinsey et al. (1999), who revealed that *Aspergillus*, *Penicillium*, and *Trichoderma* were the most prevalent genera than others in various water areas. The decomposition of fallen leaves and other detritus in streams is dominated by such fungi (Garnett et al. 2000). This is in agreement with Barron (1968), who found that *Aspergillus* is biologically one of the most successful of all fungi and expected to occur on all sorts of habitats. Besides, *Aspergillus* sp. and *Penicillium* sp. are ubiquitous saprophytes, and their spores can survive and reproduce in water (Bandh et al. 2012).

Also, El-Hissy et al. (2001) isolated 32 genera of fungi from water and mud samples in the Aswan governorate of Egypt, where the most common genera were *Aspergillus*, *Fusarium*, *Mucor*, *Penicillium*, and *Trichoderma*. In another study in Egypt, *Aspergillus* and *Trichoderma* had the highest occurrence either in surface water or submerged mud samples, and *Fusarium* was represented by low, medium, and high occurrence depending upon the tested samples, time, and place sampling (Nassar et al. 2002). Many investigators reported that *Aspergillus* and *Penicillium* have the greatest diversity and are isolated from water and sand samples collected from different areas of Egypt, Brazil, and India (Gomes et al. 2008; El-Said et al. 2010; Parveen et al. 2011; Abdel-Wareth 2014).

In the current study, *A. niger* (Asp.8) and *Penicillium* sp. (Pen. 1) were the most recorded species during summer and winter in Tanta and Giza Governorate, respectively. This result indicated that genera can tolerate extreme environmental stresses, such as high temperatures and low water availability, and can be recovered under appropriate conditions (Bandh et al. 2012). Also, Khulbe and Durgapal (1992) declared that the maximum number of fungi was during summer, autumn, and winter, which was attributed to the increase in organic matter and more feasible temperatures in these seasons.

It was found that the largest diversity of fungi was observed during spring in Tanta and autumn in Giza. These results may attributed to certain physicochemical characteristics of each area, which form suitable habitats for some fungi species. So, the high temperature recorded in Tanta during spring may have caused the tolerant fungi species to grow and distribute. Also, these results were supported by the current results of principal component (PC) analysis, which showed that temperature was positively correlated with some fungi species (Asp.2, Asp.5, Asp.7, Fus.2) and negatively correlated with fungi species (Asp.9 and Fus.1). Khallil et al. (2020) postulated that the variation in certain fungal species depends on temperature. Nassar et al. (2002) recorded that the richest samples of collected fungi from water and submerged mud were during the low or moderate temperature months. Meanwhile, Talley et al. (2002) found that temperature is negatively related to fungal richness, but not abundance. Many authors documented that the aquatic fungal communities have been affected by temperature variations in temperate regions, as temperature could be control the metabolic rates of various species and the interplay between biotic and abiotic variables (Suberkropp 1992; Wetzel 2001; Bärlocher et al. 2008; Saad et al. 2014).

On the other hand, Nasser (2005), Khallil et al. (2020), and Mustafa et al. (2023) postulated that the number of fungal species was associated with pH, DO, and TDS, which indicated that these parameters could affect the distribution and growth of fungi species. In Giza, the highest diversity of fungi species was found during autumn, indicating that the recorded species can grow at moderate temperatures, natural pH, and extremely low dissolved oxygen levels. This may be explained the strong positive correlations obtained between fungal species (Tri 1, Tri 2, and Fus. 3) and temperature (T), (Asp.5, Pen.2, Pen.4, and West.1) and DO, TDS, and EC, and (Tal.1) and pH. Abdel-Wareth (2014) recorded the highest frequency of *Aspergillus* sp. during autumn in El-Giza, El-Ismailia, and El-Menoufiya, while *Penicillium implicatum* was during summer in El-Gharbeya. Also, Jan et al. (2014) declared that the diversity of fungi species relies on the variations in physicochemical characteristics of the habitat, where the maximum numbers of *Penicillium* sp. and *Aspergillus* sp. were found during summer to early autumn and winter.

In contrast, Medeiros et al. (2009) found that the decreased oxygen levels in streams affect the diversity and activity of aquatic hyphomycetes and consequently leaf litter decomposition. Also, Asan et al. (2003) found a positive correlation between the concentration of water fungi and pH levels. Meanwhile, Abdel-Wareth (2014) revealed a positive correlation between (*Trichoderma* sp. and Phycomycetes) and TDS, (*Penicillium* sp.) and EC, and (Phycomycetes) and pH.

Regarding the significant role of aquatic plant diversity in the ecosystem, ten species were found in Giza, while six were in Tanta. *E. crassipes* and *L. gibba* were the dominant species in the investigated areas. This result agreed with the findings of Mostafa (2007); El-Deeb et al. (2017); and Al-Thahaibawi et al. (2021), who reported that *E. crassipes, C. demersum,* and *L. gibba* were the most common macrophytes present in Egypt. Also, conical corresponding analysis (CCA) showed that aquatic plants were positively associated with temperature (T) at Giza and T, EC, and TDS at Tanta. In addition to water quality parameters, the composition of substratum, climate, latitude, salinity, depth of water, eutrophication, presence of contaminants, and light can be control the growth, biomass, densities, and distribution of the aquatic plants (Smith 2004; Abou-Hamdan et al. 2005; Patil et al. 2016; Ismail et al. 2021). Furthermore, the free floating macrophytes such as *E. crassipes, C. demersum*, and *L. gibba* have a better chance to survive in polluted watercourses compared to the rooted macrophytes, where the concentrations of pesticides are generally higher in sediment than in water. Also, the floating macrophytes were found as successful bioaccumulators of numerous organic pollutants (Laet et al. 2019; Abdel-Wareth and Abd El-Hamid 2024).

Also, 10 and 9 snail species were recorded in Giza and Tanta, respectively. These species belonged to pulmonate snails *(Lymnaea natalensis*, *Succinia cleopatra, Planorbis planorbis, Helisoma duryi, Bulinus truncatus, Biomphalaria alexandrina,* and *Physa acuta*) and prosbranch snails (*Bellamya unicolor, Lanistes carinatus, Gabbiella senaariensis, Melanoides tuberculata,* and *Cleopatra bulimoides*). The same snail species were recognized by El-Kady et al. (2000); Fisher and Williams (2006); Abd El-Wakeil et al. (2013); and El-Khayat et al. (2017) in different Egyptian watercourses. *P. acuta* was the most numerous snails at all the investigated sites, indicating their ability to live in all kinds of freshwater environments (Vázquez-Capote et al. 2015), have a high capacity to tolerate great variability in abiotic factors, and successfully withstand extreme physical and chemical parameter values (Banha et al. 2014; Taybi et al. 1805). On the other hand, CCA explained that the distribution of snail species may rely on aquatic plants and variations in water physicochemical parameters. El-Deeb et al. (2017) found that sites where snails were associated with aquatic plants had higher chemical and physical parameters than sites with snails alone. Also, macrophytes play a beneficial role in improving snails’ tolerance to adverse conditions (El-Khayat et al. 2009), provide shelter and a food source (De Souza and Demelo 2012), and are used for laying snails' eggs on plant leaves (Van Schayck 1985), besides it can modify the negative effects of water pollution on snail biology, as well as physiological and behavioral adaptations (El-Khayat et al. 2011). Also, it was shown that negative associations between physicochemical parameters (temperature, D.O, pH, TDS) and snail species (*H. duryi, L. carinatus, C. bulimoides, L. natalensis,* and *S. cleopatra*) in Giza, indicating their extremely sensitive to these parameters, can cause thermal stress and reduce dissolved oxygen levels in the water (Hofkin et al. 1991; El-Khayat et al. 2009; El-Deeb et al. 2017).

Environmental factors, seasons, and pesticide application procedures can affect the concentrations of pesticide residues in water courses (Domagalski et al. 2010). In the present study, certain pyrethroid and triazole concentrations were high during spring, summer, and autumn. This finding may attributed to the excessive pesticides applied during these seasons for cultivated crops against insect attacks and bacterial and viral infections. Pyrethroids and triazoles are widely used insecticides and fungicides in many countries and are extensively used in agriculture and aquaculture (Silva et al. 2013). Besides, triazoles are known to persist in water, soil, and food because of their low biodegradability, high chemical and photochemical stability, and easy transport through the environment (Shahinasi et al. 2017). In the current work, the highest pyrethroids were deltamethrin (32.69 µg/L) and Es-fenvalerate (39.18 µg/L), lambada-cyhalothrin (29.27 µg/L) and permethrin (32.76 µg/L) in Giza, while deltamethrin (25.85 µg/L) and Es-fenvalerate (30.72 µg/L) were in Tanta. On the contrary, these results were higher than those recorded in the previous studies. Deltamethrin recorded 2.2–253 ng/L in San Diego River, Southern California (Wolfand et al. 2019), 6.28 ng/L in Beijing Guan Tin Reservoir, China, 108 ng/L in Chenab River, Pakistan, and 58.8 ng/L in Ebro River delta, Spain (Xue and Xu 2006; Riaz et al. 2018; Feo et al. 2010). Also, lambada-cyhalothrin was recorded (447 ng/L) in drains of the Salinas and Santa Maria River watershed (Anderson et al. 2018), 30.3–96.9 ng/L in the San Diego River (Wolfand et al. 2019), and 55–88 ng/L in streams in Central Germany (Bereswill et al. 2013). Meanwhile, permethrin and Es-fenvalerate ranged from trace to 0.094 µg/L in Tributaries of the Sacramento and San Joaquin Rivers, California (Bacey et al. 2005).

In the present study, tebuconazole (521.57 µg/L), tetraconazole (543.18 µg/L), and difenoconazole (413.34 µg/L) were the highest in Giza, while tebuconazole (129.39 µg/L) and tetraconazole (97.69 µg/L) were the highest in Tanta. Guarda et al. (2020) recorded tetraconazole and tebuconazole in the water of the Formoso River, Brazil, but in low concentrations as compared to those detected in our study, as the maximum concentration of tetraconazole was 0.118 μg/L, while that of tebuconazole was 0.185 μg/L. Also, in water samples from Medjerda River, Tunisia, the highest recorded tebuconazole concentration was 9.83 ng/L (Necibi et al. 2021). On the other hand, Zhang et al. (2011) recorded the maximum concentration of difenoconazole in Chinese paddy water reached 2.91 mg/L.

Concerning negative and positive correlations between pyrethroid and triazole pesticides and snails and fungi communities in the two investigated areas, these results may shed light on the ability/disability of certain snails and fungi species toward different pesticides. Al-Alam et al. (2022) documented that the diversity and distribution of snails may increase or decrease depending on the class of pollutants. Gastropods are ubiquitous in aquatic ecosystems and susceptible to various pollutants including pesticides, industrial chemicals, and metals (Alonso and Camargo 2009; Hellou 2011). El-Khayat et al. (2013) reported the spread of *B. alexandrina* and *B. truncatus* in different waterways in all centers of Kafer El-Sheikh Governorate that were contaminated with different pesticides. In addition, Becker et al. (2020) found that *Bulinus africanus* and *Biomphalaria pfeifferi* were highly tolerant to certain pesticides in different locations in Kenyan Lake Victoria. Abdel-Halim et al. (2019) and Sayed et al. (2021) found negative correlations between different pesticides and snail species in different water streams in Egypt.

Although triazoles are primarily used as fungicides, positive correlations were observed between certain fungi species and triazoles, suggesting that these fungi can tolerate high concentrations of these pesticides. These findings need further research and could explore the capacity of the tolerant fungi species as bioremediation tools (Yang et al. 2013; Bordagaray et al. 2016).

## Conclusion

The present study provided new viewpoints for evaluating the ecological threat of pyrethroids and triazoles in aquatic ecosystems. The results indicated that water quality parameters and seasonal variations could control fungal diversity and the distribution of snails and aquatic plants. The negative and positive correlations between pyrethroids and triazole pesticides and snails and fungi communities in the two investigated areas highlighted the ability/inability of certain snails and fungi species to commensalism with pesticide concentrations. Regular water source monitoring in Egypt is mandatory to get the recent status of pesticide contamination. New eco-friendly techniques should be studied to avoid reliance on chemical pesticides and legislative regulations be applied to protect aquatic ecosystems and human health from such contaminants. Further research is needed to explore the efficacy of certain fungi species as bioremediation tools and how to use different cultures and primers to cope with the bias in detecting certain taxa.

## Supplementary Information

Below is the link to the electronic supplementary material.

Supplementary file1 (DOCX 28 KB)

## Data Availability

All data generated or analyzed during this study are included in this published article.
