# Supplementary material for: Biological Diversity Associated with Pesticides Residues in Certain Egyptian Watercourses
Source: Arch Environ Contam Toxicol. 2025 May 9;88(4):419–36. doi: 10.1007/s00244-025-01129-6 (PMC12126350; doi:10.1007/s00244-025-01129-6)
Supplement: Supplementary file 1 — Supplementary file1 (DOCX 28 KB) [file 244_2025_1129_MOESM1_ESM.docx]

**Method validation**

In this experiment, we aimed to expound that our method is strongly suitable for extracting and quantitatively determining the levels of pyrethroid and triazole pesticides in water. We validated our analytical method according to **SANTE/12682/2019** guidance, which included assessing accuracy, LOD, LOQ, precision, linearity, and trueness (bias). The value of limit of detection (LOD) and the limit of quantification (LOQ) were 3.3 and 10 μg/L, respectively for the detected pyrethroid and triazole residues in water As part of the testing process, we conducted a recovery experiment to determine the accuracy of our results. We analyzed five replicates of a blank sample spiked with pyrethroid and triazole pesticides at level 0.01 mg in water samples. The **SANTE/12682/2019** document specifies that acceptable mean recoveries should fall between 70% and 120%. We used the standard deviation (RSD) for repeatability (r) to evaluate our precision. We performed a similar method on similar samples in the lab over a short period. The maximum allowable limit for the relative standard deviation of Repeatability (RSDr) was set at ≤ 20%. We tested five replicates at recovery level 0.01 mg over a day and repeated the process over three different days to ensure precision. In the current study, the limit of detection (LOD) was calculated following the equation (LOD = LOQ / 3, i.e. LOD = 10/3 = 3.3 μg/L) for pesticides in water samples (Table 1 & 2). Abdel Ghani and Hanafi (2016) found that the LODs of the tested pesticides varied from 0.3 to 4 μg/L. Also, Brondi et al. (2011) reported LODs ranging from 2 to 3 μg/L for pesticides in water samples and ranging from 3 to 20 μg/kg for pesticides in sediment samples.

**Linearity:**

To assess the method's sensitivity, we used serial standard calibration curves. However, to improve its accuracy, we implemented matrix standard calibration curves in the quantitative calculation instead of solvent standard calibration curves. We plotted standard concentrations against quantitative ion chromatographic peaks' responses to construct calibration curves. Both fungicides showed excellent linearity of the calibration curves, as evidenced by a correlation coefficient (R2) higher than 0.999. Linear regression equations were obtained using peak areas from varying concentrations of fungicides.

**Trueness and precision:**

As a part of the study, concentration of pyrethroid and triazole standard solutions were added to distilled water. The recovery mean was determined in five replicates at fortification level 0.01 mg by spiking ten ml of distilled water with the standard solution, all done by a single analyst in a day. To evaluate the accuracy of the technique, the relative standard deviation (RSD) was examined, and the observed area had an RSD of less than 20%. To assess the intra-day precision, RSDr was tested by analyzing five replicates at the LOQ level of 0.01 mg/kg on the same day. This demonstrated that pyrethroid and triazole pesticides can be identified with acceptable precision, as long as the extraction technique used has good recoveries (Table 1 & 2).

**Ion source:**

The MS detector was run in scan mode from 50 to 500 m/z, the ion source was electron ionization (EI) mode, the ion source temperature was 300°C, the quadrupole temperature was 180°C, the transfer line temperature was 280°C, and the electron energy was 70 eV. Three ions were selected for each pesticide. The highest relative abundance ion was used as the quantifier ion, while the other ions were used for confirmation as qualifier ions (Table 3).

**References**

**SANTE/12682/2019**. Guidance Document on Analytical Quality Control and Method Validation Procedures for Pesticides Residues Analysis in Food and Feed. Available online: [https://ec.europa.eu/food/sites/food/files/plant/docs/pesticides_mrl_ guidelines_wrkdoc_2019-12682.pdf](https://ec.europa.eu/food/sites/food/files/plant/docs/pesticides_mrl_%20guidelines_wrkdoc_2019-12682.pdf)

Abdel Ghani SB, Hanafi AH (2016) QuEChERS method combined with GC‒MS for pesticide residues determination in water. J Ana Chem 71: 508-512.‏

Brondi SD, De MacEdo AN, Vicente GHL, Nogueira ADA (2011) Evaluation of the QuEChERS method and gas chromatography–mass spectrometry for the analysis pesticide residues in water and sediment. Bull Environ Contam Toxicol 86: 18-22.‏

Table (1): Calibration parameters, LOQ, and accuracy of pyrethroid pesticides in water samples.

| **Pesticides** | **Linearity range (µg/L)** | **%Recovery**  ± RSD (%) | **%RSDr*** | **LOQ**  **(µg/L)** | **LOD**  **(µg/L)** | **Regression equation** | **Correlation coefficient (R^2^)** |
| --- | --- | --- | --- | --- | --- | --- | --- |
| Bifenthrin | 5000-10 | 97.49 ± 1.25 | 1.28 | 10 | 3.3 | y = 2156.7x - 78.693 | 0.9995 |
| Fenpropathrin | 5000-10 | 99..22 ±1.51 | 1.52 | 10 | 3.3 | y = 9762.9x + 1481.6 | 0.9977 |
| Lambada-cyhalothrin | 5000-10 | 98.55 ± 1.17 | 1.19 | 10 | 3.3 | y = 279.33x + 28.883 | 0.9992 |
| Permithrin | 5000-10 | 96.81 ± 1.90 | 1.96 | 10 | 3.3 | y = 1102.2x + 82.312 | 0.9989 |
| Cypermithrin | 5000-10 | 102.26± 1.14 | 1.11 | 10 | 3.3 | y = 321.6x - 16.101 | 0.9995 |
| Es-fenvelerate | 5000-10 | 97.48 ± 1.07 | 1.10 | 10 | 3.3 | y = 1125.7x + 25.844 | 0.9994 |
| Deltamethrin | 5000-10 | 100.53 ± 1.27 | 1.26 | 10 | 3.3 | y = 83.292x + 2.5097 | 0.9994 |

Intra-Day (n = 5, on one day) at the spike level of 10 μg/L. Limit of quantification (LOQ) and limit of detection (LOD) = LOQ/3.3.

Table (2): Calibration parameters, LOQ, and accuracy of triazole fungicides in water samples.

| **Pesticides** | **Linearity range (µg/L)** | **%Recovery**  ± RSD (%) | **%RSDr*** | **LOQ**  **(µg/L)** | **LOD**  **(µg/L)** | **Regression equation** | **Correlation coefficient (R^2^)** |
| --- | --- | --- | --- | --- | --- | --- | --- |
| Tetraconazole | 5000-10 | 93.34 ± 2.11 | 2.26 | 10 | 3.3 | y = 119.51x + 19.05 | 0.9993 |
| Penconazole | 5000-10 | 94.16 ±1.81 | 1.92 | 10 | 3.3 | y = 195.14x + 4.8726 | 0.9999 |
| Flusilazole | 5000-10 | 92.57 ± 1.54 | 1.66 | 10 | 3.3 | y = 158.25x + 26.603 | 0.9984 |
| Diniconazole | 5000-10 | 90.86 ± 1.44 | 1.59 | 10 | 3.3 | y = 144.74x + 6.5626 | 0.9996 |
| Propiconazole | 5000-10 | 95.94± 2.86 | 2.98 | 10 | 3.3 | y = 321.6x - 16.101 | 0.9995 |
| Epoxiconazole | 5000-10 | 95.99 ± 1.19 | 1.24 | 10 | 3.3 | y = 739.27x + 94.544 | 0.9991 |
| Tebuconazole | 5000-10 | 92.68 ± 1.84 | 1.98 | 10 | 3.3 | y = 140.28x + 33.014 | 0.9993 |
| Difenconazole | 5000-10 | 94.22 ± 1.53 | 1.62 | 10 | 3.3 | y = 168.65x - 4.5666 | 0.9996 |

Intra-Day (n = 5, on one day) at the spike level of 10 μg/L. Limit of quantification (LOQ) and limit of detection (LOD) = LOQ/3.3.

Table (3): Log *P* and monitored ions for each tested pesticides.

| **Pyrithroids** | **Log *P*^a^** | **Monitored ions^b^ (*m*/*z*)** | **Triazoles** | **Log *P*^a^** | **Monitored ions^b^ (*m*/*z*)** |
| --- | --- | --- | --- | --- | --- |
| Bifenthrin | > 6 | 181, 165, 166 | Difenoconazole | 4.2 | 265, 323, 267 |
| Cypermithrin | 6.6 | 163, 181, 209 | Diniconazole | 4.3 | 268, 70, 270 |
| Deltamethrin | 4.6 | 181, 253, 77 | Epoxiconazole | 3.44 | 192, 138, 194 |
| Es-fenvelerate | 6.22 | 125, 167, 181 | Flusilazole | 3.74 | 233, 206, 234 |
| Fenpropathrin | 6 | 97, 55, 181 | Penconazole | 3.72 | 159, 248, 161 |
| Lambada-cyhalothrin | 6.9 | 181, 197, 208 | Propiconazole | 3.72 | 69, 173, 259 |
| Permethrin | 6.1 | 183, 163, 165 | Tebuconazole | 3.7 | 125, 70, 250 |
|  |  |  | Tetraconazole | 3.56 | 336, 338, 101 |

^a^ Octanol–water partition coefficient. ^b^ The 1^st^ ion m/z was used for quantitation, while the other ions were used for confirmation ions.
